# Management of chronic kidney disease: perspectives of Brazilian primary care physicians

**DOI:** 10.1017/S1463423621000074

**Published:** 2021-03-17

**Authors:** Thatiane Delatorre, Elen A. Romão, Augustus T. R. de Mattos, Janise B. B. Ferreira

**Affiliations:** 1 Social Medicine Department of the Medical School of Ribeirão Preto, Universidade de São Paulo, Ribeirão Preto, SP, Brazil; 2 Medical Clinic Department of the Medical School of Ribeirão Preto, Universidade de São Paulo, Ribeirão Preto, SP, Brazil; 3 Medicine Department of Universidade Federal de São Carlos, São Carlos, SP, Brazil

**Keywords:** chronic kidney disease, primary care physicians, primary health care

## Abstract

**Aim:**

To investigate primary care physicians’ knowledge of and attitudes toward care for chronic kidney disease patients.

**Background:**

In Brazil, care for chronic kidney disease, a global public health problem, is provided by the Brazilian National Health System, which is organized around primary care. The study aimed to investigate the knowledge and attitudes of primary care physicians about the management of chronic kidney disease.

**Method:**

This research is based on quantitative and qualitative data. The participants were 92 physicians from 81 primary care units located in eight cities of the São Paulo/Brazil health region, who answered a self-administered questionnaire.

**Findings:**

Only 59% and 58% of the physicians recognized smoking and obesity, respectively, as risk factors for chronic kidney disease. Health appointments and drug therapy predominated as disease prevention strategies and less than 30% mentioned multiprofessional care and health education groups. For early diagnosis, isolated serum creatinine was the most used test and 64.6% stated they classified the disease stages. Exclusive follow-up in primary care decreased from 79% in stage 1 to 19.5% in stage 3B and the patients’ monitoring in the healthcare network varied from 8.7% in stage 1 to 70.6% in stages 4 and 5ND, suggesting early referrals and lack of referral at the necessary stages. Access to information on the referred patient was, predominantly, through the patient’s report and 74% of the physicians did not have matrix support regarding chronic kidney disease.

**Conclusion:**

The study showed that the healthcare teams need to update their knowledge and procedures to be able to provide a comprehensive and efficient approach to treating chronic kidney disease in primary care.

## Introduction

Chronic Kidney Disease (CKD) is defined as abnormalities in the kidney structure or function, which are present for more than three months and with health implications. The criteria used to define CKD are the presence of markers of kidney damage for more than three months (ie, albuminuria, urine sediment abnormalities, electrolyte and other abnormalities due to tubular disorders, abnormalities detected by histology, structural abnormalities detected by imaging, and history of kidney transplantation) and/or the glomerular filtration rate (GFR) below 60 mL/min/1.73 m^2^. The CKD classification is done based on the cause of kidney disease, GFR category, and albuminuria category.

By associating GFR-based categorization with albuminuria, categories with a similar relative risk for CKD outcomes are obtained. Thus, CKD is divided into six stages of GFR (1, 2, 3A, 3B, 4, and 5) and three proteinuria stages (1, 2, and 3) with stage 1 representing normal kidney function and category 5 representing kidney failure (KDIGO, 2013 ).

CKD is a global public health problem that currently affects more than 750 million people. In 2017, it was estimated that the prevalence of any stage of CKD among Medicare patients in the American population was 14% (the United States Renal Data System, 2019). In Brazil, the prevalence of CKD is not known (Marinho *et al.*, 2017). It is estimated that in 2017 the prevalence rate of kidney failure treated with replacement therapy was 610 patients per million (ppm) (Thomé *et al*., 2019).

The impact of kidney disease, its diagnosis, and treatment vary in different parts of the world, and its magnitude is better known in the developed countries.

However, recent evidence suggests that its effects are similar or may have greater importance in the developing countries (GBD 2015 DALYs and Collaborators HALE, 2016; Crews *et al*., 2019).

Robust scientific data indicate that CKD progression is linearly associated with worsening outcomes (Levey *et al*., 2011). The identification of CKD in its early stages is essential to prevent its progression, morbidity, and mortality, as well as to reduce the healthcare expenditure of persons with CKD. Therefore, public health approaches that enable the early initiation of treatment and mitigation of risk factors for the progression of this disease are essential (Vivekanand *et al*., 2013; United States Renal Data System, 2019).

Despite insufficient data that fail to reflect the real impact of kidney disease, Brazil is one of the 21% of the world’s middle-to-low income countries that have government funding for all aspects of CKD treatment through its National Health System (SUS), which is a universal health care system (Crews *et al*., 2019; GBD 2015 DALYs and Collaborators HALE, 2016).

Several studies warn of the financial impact of CKD on public and private health care systems. Spending on kidney failure with replacement therapy represents about 5% of SUS spending on medium and high complexity. In addition to government and personal assistance expenses, some losses are difficult to measure, since CKD also causes social and emotional repercussions in the life of the affected person and family members. These repercussions come from the chronicity, evolution, and complications of the disease and its treatment that affect the quality of life; physical, work, and functional capacity; sexual health; and leisure of the patients (Moreira *et al*., 2016; Alcalde and Kirsztajn, 2018; Silva Junior *et al*., 2018).

The central locus of care for chronic conditions in the SUS is primary care, a structure with large capillarity that is the gateway of the health care network. Primary care offers a broad set of individual and collective actions to solve the most common health problems (Starfield, 2002; Ministério da Saúde-BR, 2010; Mendes, 2012; Ministério da Saúde-BR, 2017).

In the world’s political agenda, the importance of kidney disease has not yet been widely recognized, which makes it a neglected disease. The Global Action Plan for the prevention and control of non-communicable diseases (NCDs) of the World Health Organization (WHO) does not include kidney disease. However, it can contribute to more deaths, in the world, than the four main NCDs together (Crews *et al*., 2019).

Thus, considering CKD’s impact on developing countries, the role of primary care, and the coverage of SUS, this study aimed to investigate primary care physicians’ knowledge and attitudes concerning care for CKD patients.

## Methods

This research is based on quantitative and qualitative data. The participants were 92 physicians (50.3%) out of 183 physicians working in 81 primary care units in eight cities of a health region of the state of São Paulo, Brazil, with a population of approximately 900,360 inhabitants (IBGE, 2017). An initial survey was carried out through the Ministry of Health’s National Health Establishment Registration System (SCNES) to define the number of participants. After this stage, the researchers contacted the municipal managers of the primary health care (PHC) units of the studied health region by email and/or telephone, with the objective of scheduling visits to the primary health care units to explain the research to physicians and collect data. To be included in the study, the physician must have been working in primary care for at least one year. The exclusion criteria adopted were less than one year of working in primary health care, being on vacation, or away from work at the time of data collection. Data were collected from May to November 2017. The participants answered a self-administered questionnaire that had been previously submitted to a content validation process by six expert medical reviewers experienced in the subject, and circulated in the clinical practice of the national health services (Supplementary file 1). The content of the questionnaire was consistent with the federal clinical guidelines for CKD care, with the Brazilian Ministry of Health’s strategic action plan to manage chronic non-communicable diseases (CNCDs) and with the Brazilian laws that regulate the organization of this line of care (Ministério da Saúde-BR, 2011; 2014a; 2014d). It contained semistructured questions divided into four dimensions: Dimension 1—clinical guidelines for the care of patients with CKD in SUS and within PHC (identification of risk groups, prevention strategies for CKD in patients at risk of developing the disease, and early diagnosis and treatment); Dimension 2—monitoring of the patient in the care network of patients with CKD; Dimension 3—availability of resources in the line of care for patients with CKD; and Dimension 4—coordination of care for people with CKD within PHC. It also contained an open question: Would you like to write about some difficulty you have experienced in assisting CKD patients in primary care? (Supplementary file 2).

The data were not recorded, as the participants completed a questionnaire with the majority of structured questions and one unstructured question. The quantitative data collected were entered into the public domain in Epi Info™ 7.2, the Centers for Disease Control and Prevention (CDC) computer program, and were analyzed using descriptive statistics. In contrast, the qualitative data obtained from the questionnaire’s unstructured question were transcribed and subjected to thematic analysis, consisting of phases such as pre-analysis, material exploration, and treatment of results, inference, and interpretation (Bardin, 1977).

Ethics review and approval for our evaluation of these data was received from the institutional review board of the Centro de Saúde Escola, Faculty of Medicine of Ribeirão Preto, University of São Paulo.

## Results

The findings revealed that the majority of the 92 participants were women in the age group 31–40 years, with average education duration, for the physicians who provided this information (*n* = 70), of 14.2 years (Table 1).

**Table 1.** General profile of the medical professionals who participated in the study, 2017

Variable		Physician	
		N	%
Sex	Female	47	51.1
	Male	45	48.9
Age group	25–30	8	8.7
	31–40	41	44.6
	41–50	15	16.3
	51–60	14	15.2
	60 and older	3	3.3
	Not informed	11	11.9
Nationality	Brazilian	81	94.2
	Cuban	3	3.5
	Bolivian	1	1.2
	German	1	12
Postgraduate education	Residency	62	67.4
	Specialization	43	46.7
	Master’s degree	8	8.7
	Doctoral degree	6	6.5
	Others	10	10.9
Year of graduation	1972–1979	2	2.2
	1980–1989	6	6.5
	1990–1999	13	14.1
	2000–2009	28	30.4
	2010–2016	21	22.8
	Not informed	22	23.9

Among the participants with medical residency (*n* = 62; 67%), 20 (32%) had completed residency programs in a medical clinic and two (3.2%) in nephrology. The second most prevalent specialty was family and community medicine (*n* = 19, 30%) (Table 1).

Half (*n* = 46, 50.5%) of the physicians reported not being familiar with the current law about the provision of assistance to CKD patients in primary care, but 83% (*n* = 76) considered themselves ‘capable’ of dealing with CKD in the stages attributable to primary care. In the ‘not capable’ answers given by 17% (*n* = 16) of the participants, they emphasized the gaps in health education.

Of the 87 physicians who responded about having received training to treat CKD, only 42.5% (*n* = 37) said yes. Of these, 25 (67.5%) reported the year of training, with the median time between the year of graduation and the year of training being 14 years (range 1–35 years).

Table 2 presents the data about participants’ knowledge of risk factors for the development of CKD, the predictive factors (markers that give worse prognosis for loss of renal function), the offerings of health actions for the care of population groups at risk of developing CKD and the referral of cases to specialized care. Uncontrolled hypertension and blood glucose were identified as the main predictive factors.

**Table 2.** Risk factors, predictive factors of prognosis, health actions offered to patients, and characteristics of referrals of CKD cases to specialized care, 2017

CKD risk factor	Number of participants*	%
Arterial hypertension	92	100
Diabetes	92	100
Nephrotoxic agents	85	92
Circulatory system disease	79	
CKD in the family	72	78
Age	66	72
Smoking	54	59
Obesity	53	58

Prognostic predictor factor	Number of participants	%
Poorly controlled blood glucose	91	98.9
Arterial hypertension	91	98.9
Albuminuria intensity	82	89.1
Nephrotoxic agents	80	86.9
Advanced stages of CKD	67	72.8
High cholesterol	49	53.2
Smoking	45	48.9
Others	3	3.2

Health action offered	Number of participants	%
Drug treatment	88	96
Health consultation	75	82
Home visits	47	51
Multiprofessional service	25	27
Health education groups	24	26

Characteristics of CKD referrals to specialized care		
CKD staging	Numberº of participants	%
G1	8	8,7
G2	16	17,3
G3a	35	38,0
G3b	62	67,3
G4	65	70,6
G5 (non-dialysis)	65	70,6
Grouped analysis: G1 to G3b	75	81,5
Other criteria	25	27,0

Knowledge of the clinical guidelines*	Number of participants	%
Know and use	70	77,0
Unknown	21	23,0

Regarding the time to carry out the referral**	Number of participants	%
Unknown	46	50,0

* Clinical guidelines for the care of patients with chronic kidney disease in SUS, 2014.

** Consultation with the nephrologist

CKD = chronic kidney disease

Regarding home visits, 51% (*n* = 47) of doctors mentioned that this offer was available at the health service (Table 2). Home visits, in the view of these 47 participants, were performed more frequently by nurses (*n* = 33, 70%), doctors (*n* = 33, 70%), and community health workers (CHA) (*n* = 30, 63%). Of the 61 educational activities cited by 26% (*n* = 24) of doctors, the most mentioned themes were healthy eating (*n* = 24, 100%) and arterial hypertension (AH) and/or diabetes mellitus (DM) (*n* = 19, 79%).

The time described by the participants (*n* = 46, 50%) between referral and consultation with the nephrologist was three months (range: 1–12). Thirty percent (*n* = 27) of the participants reported not referring patients in stages 4 and 5 to nephrologists.

Integration of primary care and the specialized nephrology services of the health care network was mentioned by 66% of the physicians (*n* = 61). As for secondary and tertiary specialized CKD services available in the city where the physician worked, 61% (*n* = 56) of the physicians reported that their city offered the services; 19.5% (*n* = 18) said their city did not have these resources; and 19.5% (*n* = 18) did not know whether the city offered the resources. The majority (*n* = 67, 73%) of the 91 participants who answered this question reported not receiving matrix support or consultancy regarding CKD. In comparison, 16% (*n* = 15) said they receive it informally by contacting colleagues, and only 10% (*n* = 9) reported receiving it formally from the institution where they work.

When the user was referred to another care provision unit of the health care network to receive a follow-up, 80% (*n* = 73) of the participants (*n* = 91) reported that the PHC health team accessed information predominantly through the patient’s report (65%, *n* = 59), or through an electronic information system (32%, *n* = 29), and only 20% (*n* = 18) through physical counter-reference. Still, 94% (*n* = 86) reported that the referred patient maintained regular return visits to PHC.

The majority of the physicians (*n* = 74, 80.4%) reported requesting clinical laboratory tests (Table 3) of patients at risk of developing CKD; 7.6% (*n* = 7) of the physicians mentioned requiring tests of all patients, regardless of the presence of risk factors; and 11.9% (*n* = 11) mentioned other criteria to request tests. Concerning the average time elapsed between the request of the test and the patient’s receipt of the result, most of the physicians (*n* = 83, 91.2%) knew about it, with a median of 60 days (variation: 2–182).

**Table 3.** Diagnostic resources used in the identification of CKD by primary care physicians, 2017

Laboratory test	N	%
Serum creatinine	82	89%
GFR serum creatinine	70	76%
Urinalysis (urine spot sample)	66	72%
Ultrasound kidneys and urinary tract	63	68%
Albumin-to-creatinine ratio	53	58%
24-hour microalbuminuria	49	53%
GFR 24-hour urine collection	23	25%
Isolated microalbuminuria	17	18%
24-hour creatinine clearance	11	12%
X-ray urinary tract	7	8%
Other resources	4	4%
Computed tomography	2	2%

GFR = glomerular filtration rate.

In the cross-analysis, we found that 23% (*n* = 19) of the professionals who used serum creatinine did not use the GFR from serum creatinine. The combined use of GFR from serum creatinine and urinalysis (urine spot sample analysis) was employed by 56% (*n* = 52) of the physicians for the diagnosis of CKD.

As for the periodicity with which the physicians requested tests of at-risk patients in whom CKD was not identified in the first assessment, 37% (*n* = 34) requested tests once a year, followed by 26% (*n* = 24) who requested them every semester. Furthermore, 89.1% (*n* = 82) reported assisting the patient with CKD in the primary care service where they worked, and 64.6% (*n* = 53) reported classifying the CKD stages.

**Figure 1.**
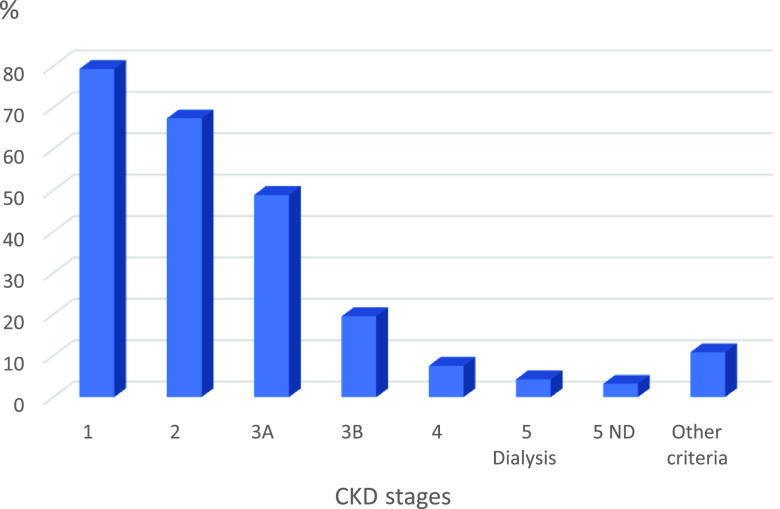
CKD follow-up in primary care according to classification stages, 2017

The diagnosed patients’ follow-up exclusively in primary care varied according to the stages (Figure 1): 79.3% (*n* = 73) of physicians only followed stage 1 patients; 67.3% (*n* = 62) followed the patients until stage 2; 48.9% (*n* = 45) until at stage 3; and 19.57% (*n* = 18) until at stage 3B. Only 10 physicians (10.8%) reported following up patients at stages 1, 2, 3A, and 3B exclusively in primary care.

Regarding the objectives of the clinical management of CKD patients in primary care, despite the high frequency (close to or above 90%) of all the goals pertinent to primary care, smoking cessation and stimulus to physical activity were the least cited (*n* = 80, 87% and *n* = 83 90%, respectively).

Table 4 presents the thematic analysis of the physicians’ statements on the capacity and the main difficulties in the approach to CKD in PHC, according to the main themes, frequency of sense nuclei, and exemplification of the statements.

**Table 4.** Frequency and examples of physicians’ discourse, according to the sense nuclei and on the ability and difficulties to address CKD in primary care, 2017

Capability for dealing with CKD in the stages attributable to Primary Care according to physicians			
Sense nuclei	Example of the participant’s discourse	Number of nuclei	%
Health education	‘Because I haven’t learned to follow up severe kidney patients adequately. I want to have continuing education about the theme’. ‘More training is necessary’.	9	52.9
Clinical management	‘I think the theme is very complex, I just focus on prevention of risk factors’.	3	17.6
Regulatory instruments	‘There is no protocol for us to follow’.	2	11.8
Matrix support	‘Matrix support must improve so that we can provide care with better quality’.	2	11.8
Diagnostic resources	‘Because we don’t have all the laboratory diagnosis resources. The request for creatinine clearance was denied’.	1	5.9
**Total**		**17**	**100**

Main difficulties reported by physicians to approach CKD in PHC				
Theme	Sense nuclei	Examples of the participants’ discourse	N Nuclei	%
Characteristics of the care model	Health education	‘Lack of knowledge about the matter’ –M17	7	44
	Continuity of care	‘Due to the overcrowding and overloading of the primary level, there are management difficulties with premature return’. – M23	5	31
	Health team	‘The frequent absence of the multidisciplinary team, (scarce) qualification courses/professional recycling activities. Inefficient services in terms of prevention’. – M30	2	12.5
	Infrastructure	‘The difficulties are related to the services’ structure, volume, and lack of training’. – M29	2	12.5
**Total**			**16**	**100**
Chronic kidney disease care in the primary level	Clinical management	‘Indiscriminate use of anti-inflammatory drugs by patients with systemic arterial hypertension, diabetes mellitus, obesity always calls our attention during assistance. Patients are poorly informed about the pathology, adequate diets, comorbidities control. It is very difficult for the physician to approach all these things and provide guidance in a routine appointment due to lack of time’. – M71	7	58
	Diagnostic support	‘Permission to request some tests: PTH (parathyroid hormone), NTD* (with the physicians’ justification) to better follow-up stage-3A patients that could be receiving follow up only at the Primary Care Unit/Family Health Unit’. – M92	5	42
**Total**			**12**	**100**
Primary Care and Assistance Regulation in the Healthcare Network	Regulatory instruments	‘Long period of time elapsing before the patient is seen by a specialist; excessive bureaucracy in the protocol; electronic refusal without contact with the professional who referred the patient (rejection of referral); lack of integration between specialist professionals and primary care physicians to establish referral flows and CKD qualification; frequently, the specialist physician’s medical assistance is not entered into the electronic system, either because the professional does not do it or because the service does not have access to the patient’s electronic record; primary care physicians’ difficulty in following up patients – even though they are qualified, they do not have easy access to diagnosis resources that can be used exclusively by specialist physicians. This locks the system, generates queues and reduces the service’s efficiency and capacity to solve health problems’. – M76	18	50
	Integration and communication	‘Lack of matrix support by nephrology teams; defective counter-referral system that offers only partial information frequently disconnected from primary care’. – M47	11	30.5
	Accessibility	‘Difficulty in tests, both laboratory and imaging ones; lack of medicines in the healthcare network; difficulty in making an appointment with a specialist, even for assessment and guidance; long period of time elapsing before the follow-up appointment’. – M5	4	11
	Coordination of care	‘We don’t know what happens to the patient in the specialized care. It would be interesting to have these orientations for the coordination of actions in primary care’. – M57	3	8.5
**Total**			**36**	**100**

* Enzyme 5 Nucleotidase

## Discussion

This study showed a critical knowledge gap regarding the approach to CKD, as, among the group studied, half of the physicians were unaware of the national guidelines on CKD, and most professionals reported not having received training after hiring, which can interfere not only in the management but also in the access of people with this condition to care in PHC.

Research conducted in India identified several barriers to access for patients with CKD who needed urgent attention in PHC, recommending the creation of screening programs and specific educational initiatives to improve awareness of this disease. The study also points out that the PHC infrastructure needs to be strengthened for the care of patients with CKD, ensuring trained staff, availability of diagnoses, essential drugs, and creating efficient reference paths for quality care (Jafar *et al*., 2020).

The Pan American Health Organization emphasizes the world’s shortage of health workers qualified to meet the population’s health needs. Furthermore, the majority of today’s health workers were trained to treat acute diseases, not chronic problems, which require different skills and competencies (OPAS, 2015).

In Brazil, risk factors for CNCDs that contribute to the triple burden of disease predominate (Banco Mundial, 2005). Although most physicians reported feeling capable of dealing with CKD in primary care, the data showed that with respect to the prevention and strategies to slow the progression of CKD, almost none of them recognized the offering of educational actions focusing on modifiable risk factors as the first strategy of approach. A study (Silva and Brune, 2011) with patients at risk of developing CKD showed that 50% of them were in stages 3 and 4, which reveals the need for recognition and action by the primary care team about the risk factors. The physicians recognized systemic AH and DM as the main risk factors for CKD, but not all of them mentioned smoking, obesity, and sedentariness, which reflects the little importance they give to education strategies about the modifiable risk factors (Silva Junior *et al*., 2017). It is important to note that according to the project guidelines of the Brazilian Medical Association, massive proteinuria, severe AH, inadequate glycemic control in diabetics, smoking, and obesity are considered risk factors that worsen kidney injury and accelerate the fall in GFR after the onset of kidney injury (Kirsztajn *et al*., 2011).

It was noted that less than 30% of the physicians recognized multiprofessional involvement and educational groups as health care offerings for patients at risk of developing CKD. However, primary care is the most suitable locus of attention for these actions. It needs to be consolidated not only as a gateway to the health care system but also as the most appropriate place for this type of approach, through coordinated and continuous care. Thus, most CKD patients can be treated in PHC through ongoing follow-up to identify those at a high risk of progression to advanced stages, including end-stage kidney disease (Grill and Brimble, 2018).

Greer *et al*. (2012) indicate the need for investment in the educational initiatives about CKD in PHC, including the approaches taken by multidisciplinary teams, especially guiding self-care and spreading patient knowledge about the disease. Thus, the primary care team’s training on educational aspects in the management of chronic renal patients can provide qualified support to patients and families for the management of their condition and, ultimately, improve the clinical results of these patients. In this sense, a study carried out with chronic renal patients undergoing hemodialysis sought to understand how they performed self-care. From the patients’ statements, three categories emerged: requirements for self-care, self-care deficit, and education and information management for self-care. The study revealed difficulties in implementing self-care by these patients regarding resistance to changing life habits, adherence to treatment, and lack of information about their disease and treatment, as well as situations aggravated by the unfavorable economic conditions of some patients. On the other hand, the study pointed out that although the participants affirmed that they did not carry out self-care actions rigorously they were aware of the importance of carrying them out. Still, due to some limitations, it was noticed that people’s knowledge about their condition was generally acquired through the Internet and their own experience, and not through the health team. The authors concluded that people have the responsibility of maintaining their health but the health care team should recognize the conditions that interfere in these patients’ self-care to collaborate in overcoming the difficulties (Santana *et al*., 2020).

Care provision for CNCDs demands the transformation of reactive health systems into proactive systems, aiming to maintain the person as healthy as possible (OPAS, 2015). Thus, preventing CKD involves treating and controlling modifiable risk factors, and primary care must perform health actions beyond isolated and drug-based medical approaches (Ministério da Saúde- BR, 2014c; Azevedo *et al*., 2018). The importance of multiprofessional action was shown by a study in which clinical and laboratory parameters of patients with CKD improved significantly, contributing to reduce the progression of CKD (Luciano *et al*., 2012).

Results of a systematic review that sought to identify barriers and facilities that family doctors face when diagnosing and managing CKD suggest the need for time-efficient strategies that promote collaboration between members of the health care team and practical guidelines that consider the nature of CKD and the commonly associated comorbidities. A collaborative relationship between the family doctor and the nephrology services can also offer significant support to the PHC teams when diagnosing CKD, thus facilitating the patient’s self-management (Neale *et al*., 2020).

About the availability of resources for CKD treatment, many countries still do not have access to primary diagnoses and nephrology-trained workers (Crews *et al*., 2019). The approach to this chronic condition in primary care is grounded on its potential for solving health problems, based on its clinical and care capacity and on the incorporation of soft, soft-hard and hard technologies (diagnostic and therapeutic). The use of risk stratification by a little more than half of the physicians to identify subgroups according to the complexity of the chronic health condition helps in the differentiation of clinical care and of the flows that each user must follow in the health care network, for an integrated approach and rational use of resources (Mendes, 2012; Ministério da Saúde-BR, 2017).

The use of estimated GFR (eGFR) from serum creatinine associated with the assessment of albuminuria through the albumin-to-creatinine ratios (ACRs) is currently the best method to diagnose and categorize CKD (KDIGO, 2013). However, it is still neglected by a large number of the physicians in this study. GFR associated with spot urine samples analysis for the diagnosis was used by a little more than half of the physicians, although it is indicated as the first choice exam in the Brazilian clinical guidelines for caring for patients with CKD.

When the diagnosis was not made in the first screening, the great majority of them did not repeat the investigation with the recommended regularity. Evidence has shown (Silva and Brune, 2011) that the majority of patients in CKD stages 1 to 3 present serum creatinine within reference values (sensitivity of only 21% to detect individuals with altered GFR). Moreover, of the patients in stages 3 and 4, 79% present normal serum creatinine. Therefore, focusing exclusively on serum creatinine levels to evaluate GFR can delay the diagnosis of CKD. A spot urine samples analysis looking for urine sediment abnormalities that precede the reduction of GFR should always be on the minds of physicians as also ACRs, a marker of kidney damage, especially in at-risk persons such as diabetics and hypertensive patients.

Health managers and professionals must work in a concerted fashion among the target population from screening, to early diagnosis, and to appropriate diagnostic resources and the right time to use them in order to avoid inefficiency and poor care.

According to Grill and Brimble (2018), CKD screening should be performed only in patients with known risk factors and in the absence of an acute disease. The tests of choice for diagnosing CKD include GFR and spot urine ACRs. Most CKD cases in PHC have a low risk of progression and can be managed exclusively by family doctors. For patients with CKD who progress to more advanced stages or meet the reference criteria, it is essential to seek help from a nephrologist and work together to provide patients with the best care (Grill and Brimble, 2018).

The findings showed that 26% of the physicians referred patients at a very early phase (stages 1 and 2), and half of them referred them at stage 3A. On the other hand, 30% of the interviewed physicians followed up stage 4 patients in primary care.

In agreement with Vassalotti *et al*. (2016), the main reasons for referring patients with CKD to nephrology specialists are eGFR <30 mL/min/1.73 m^2^, severe albuminuria, and acute kidney injury. The ultimate goal of CKD management is to prevent disease progression, minimize complications, and promote quality of life.

The SUS utilizes a protocol (Ministério da Saúde - BR, 2014a; 2014d.) recommending that patients in stages 1, 2, 3A, and 3B should be assisted, preferably in primary care. However, the findings showed that although the majority of the physicians know and use the protocol of referral to nephrology, there was a decline in the follow-up at primary care as the stage advanced, suggesting that these physicians also referred primary care patients. The physicians frequently reported difficulties in the clinical management of CKD because it is a complex condition to approach.

In the view of patients and PHC professionals, significant limitations for care in CKD were the lack of knowledge and awareness about this disease. Barriers at the health system level included shortages of qualified professionals, medicines, fragmented referrals, and care and inadequate follow-up by specialists in hospitals. For patients and health care professionals, access to care in CKD can be improved through educational initiatives to raise awareness about CKD by the health care team in patients and their families by providing supplies and coordinating the care and involving the community health workers providing in-home care (Jafar *et al*., 2020).

Incomplete referrals or referrals at stages that could be treated in primary care and late diagnoses and referrals produce a negative repercussion for these patients’ morbidity and mortality. Adequate referral, in turn, can result in efficiency in the health care system, improvement in patients’ survival, delay in the need to begin Renal Replacement Therapy  (RRT), timely intervention for making permanent access, better treatment choices and options, reduction in the number of emergency dialyses better nutritional patterns for patients and fewer hospitalization days (Bahiense-Oliveira et al., 2010; Coutinho & Tavares, 2011; Diegoli et al., 2015; KDIGO, 2013; McLaughlin et al., 2001; Padovani et al., 2012; Peña et al., 2006; Pena et al., 2012 and Nunes et al., 2014). The adoption of regulatory and care protocols guides the requesting professionals’ decisions and modulates the evaluation performed by the monitoring physicians (Ministério da Saúde- BR, 2017). Although the implementation of regulatory protocols can cause an increase in the demand for nephrologists, joint initiatives between specialists and primary care physicians can improve referral and optimize the use of the available resources, contributing to better clinical outcomes (KDIGO, 2013). In Brazil, this joint action is recommended by the matrix support strategy, which aims to ensure specialized back-up and technical-pedagogical support to primary care teams (Campos and Dominitti, 2007; Ministério da Saúde- BR, 2014b). However, in this study, integration occurred only in two-thirds of the situation, and the matrix support provided by specialists was rarely available. Electronic support for clinical decisions is also a robust back-up and management strategy for primary care teams (Litvin *et al*., 2016).

As for the relation to other units of the health care network, the findings revealed the fragility of counter-referral and the difficult access to the referred patient’s information, which corroborates the results of other studies (Almeida *et al*., 2010; Magalhães Junior and Pinto, 2014). This situation negatively affects care coordination, an attribute of primary care inseparable from the health care network’s horizontal and vertical integration strategies, and organization of the health care network (Giovanella, 2014).

Knowledge about the health care network and its appropriation by its professionals, the network’s effective regulation, clear protocols and flows, and efficient communication among the different care provision units are necessary conditions for dealing with CKD. Such an organizational network postpones the onset of dialysis treatment in urgent and emergency care hospitals, even for CKD patients who were being followed up by a nephrologist at an outpatient clinic (Ferreira, 2015).

Another important tool to reduce fragmentation is the clinical information system, as it organizes data on individual patients and entire clinical populations (OPAS, 2015). However, in the studied scenario, the health care network showed up fragmented, poor communication, which can interfere in the occupation of vacancies in the specialized service, often by patients referred early, overloading the service and attention. Thus, PHC teams must act in an articulated manner with nephrologists from specialized services to uplift the care and life of people living with CKD (Grill and Brimble, 2018).

## Conclusion and implications

There are gaps in many countries related to awareness-raising, workforce qualification, and improvement in the assistance provided for CKD patients. In Brazil, the structure of SUS, grounded on universal access and equity, enables us to deal with the entire spectrum of CKD, from screening and preventive strategies in at-risk patients to the provision of highly specialized care, such as RRT and transplant.

However, the data showed that, especially in PHC, there is a need to qualify the care of patients at risk and patients with CKD, through the institution of relatively simple measures such as the appropriate use of available diagnostic resources and risk stratification, improvements in the connection in the health care network, provision of matrix support by specialists in nephrology, continuing education for professionals, and strategies that promote self-care by patients.

It is also necessary to enhance offerings not centered on medical care and drug therapy and to implement prevention strategies that can achieve integration of care and improvement in the management of this complex morbidity.

Indeed, investment in these aspects will facilitate better indicators related to CKD treatment in primary care, which will positively affect the patient and the health care system.

## Supplementary material

For supplementary material accompanying this paper visit http://dx.doi.org/10.1017/S1463423621000074. click here to view supplementary material
