# Supplementary material for: Management of chronic kidney disease: perspectives of Brazilian primary care physicians
Source: Prim Health Care Res Dev. 2021 Mar 17;22:e8. doi: 10.1017/S1463423621000074 (PMC8060812; doi:10.1017/S1463423621000074)
Supplement: Supplementary file 1 [file phcsup.zip › S1463423621000074sup001.docx]

**Supplementary file 1:** Questionnaire content validation process by medical judges

The proposed content validation consisted of determining whether the content of the measurement instrument effectively explores the requirements for measuring the phenomenon to be investigated. The participants in this process were instructed on the objectives of the tool, how it would be applied, and the theoretical frameworks used for its elaboration. The judges were asked whether the instrument as a whole was easy to understand (from a grammatical, semantic point of view), whether it was objective (allowing a response without subjective judgment), whether it was pertinent within the theme (relationship with the theoretical references used in the construction of the instrument and the relevance of the theme), if the instrument's scope includes all the essential questions within the PHC's attributions, in the CKD care line; if the theoretical-scientific basis used is sufficient and up-to-date, if there would be necessary but absent items in the instrument and there would still be unnecessary items in the instrument. Also, for each dimension of the tool, the judge was asked whether the questions related to the dimension were elaborated in a clear and easy to understand manner, whether they addressed all important aspects within the dimension and whether the possible answers would be adequate to the content of the question.

The medical judges (n = 6) who participated in this process were four females (66.6%) and two males (33.3%), and an average age of 45 years. The training time of 66.6% (n = 4) was over 16 years, two professionals (33.3%) had a master's and a doctorate in nephrology, two professionals had a master's (33.3%), one professional ( 16.6%) had a Master in Business Administration (MBA), two reported residency in nephrology/family medicine (33.3%), and one professional (16.6%) did not specify the type of post-graduation they had, referring only to the medical / nephrology clinical area. As for the current workplace, five professionals (83.3%) worked in clinical practice in hospitals and/or nephrology clinics, 3 of which (50%) also worked in teaching. One professional (16.6%) worked in teleconsulting. Regarding PHC experience, two professionals (33.3%) reported the experience as a network professional, two as a teacher (33.3%), 1 in other activities (16.6%), and one reported having no experience in PHC (16.6%). The validation process was vital for the improvement of the questionnaire. The contributions consisted of inclusion and exclusion of variables, adjustment of variables for better statistical analysis of the data, orthographic and semantic corrections, and changes in the order of the questions.
