# Supplementary material for: Management of chronic kidney disease: perspectives of Brazilian primary care physicians
Source: Prim Health Care Res Dev. 2021 Mar 17;22:e8. doi: 10.1017/S1463423621000074 (PMC8060812; doi:10.1017/S1463423621000074)
Supplement: Supplementary file 1 [file phcsup.zip › S1463423621000074sup002.doc]

**Data collection instrument for medical health professionals in Primary Health Care (PHC).**

| **CHARACTERIZATION OF PARTICIPANTS** | | |
| --- | --- | --- |
| DATE |  | |
| INITIALS |  | |
| DATE OF BIRTH |  | |
| SEX | ( ) F ( ) M | NATIONALITY: |
| GRAD YEAR |  | |
| POST-GRADUATE | ( ) Specialization ( ) Master ( ) Doctorate  ( ) Medical Residence, which one:  ( ) others, which one:  AREA (s):  AREA: | |
| Time in Primary Health Care (PHC) | ____Years (s) ____months | |
| Weekly workload at PHC | __________hour/week. | |
| Current Unit Type | ( )Basic Health Unit (UBS) without Family Health  ( ) UBS with Family Health  ( ) UBS with the Community Health Agents Program (PACS)  ( ) Family Health Unit (USF)  ( ) District Basic Health Unit (UBDS) with Primary Care  ( ) UBS with Parameterized Primary Care Teams | |
| Have you received any training / capacity building on Chronic Kidney Disease (CKD)? | ( ) No  ( ) Yes, year:  ( ) Offered by the service/institution that operates or has acted  ( ) Search by own means  Areas covered in the training: ( ) prevention, ( ) diagnosis, ( ) treatment of CKD,  ( ) treatment of risk factors that lead to CKD, ( ) other topics, which one: _______________. | |

**Important guidelines:**

The questions below refer to care for patients with chronic kidney disease in the context of Primary Health Care;

**It is requested not to carry out consultations to answer them;**

You can tick one or more alternatives in each question, as you judge the correct answer.

| **DIMENSION 1- CLINICAL GUIDELINES FOR THE CARE OF CHRONIC KIDNEY DISEASE - DRC IN**  **UNIQUE HEALTH SYSTEM (SUS) WITHIN THE FRAMEWORK OF PRIMARY HEALTH CARE.** | | | | | |
| --- | --- | --- | --- | --- | --- |
| **SUBDIMENSION 1.1: Identification of risk groups.** | | | | | |
| Q1- Which individuals do you consider at risk of developing Chronic Kidney Disease? | - People with diabetes (whether type 1 or type 2) - Hypertensive people - Elderly (> 60 years) - Obesity patients (BMI> 30 Kg / m²) - History of circulatory system disease (coronary heart disease, stroke, peripheral vascular disease, heart failure) - the history of CKD in the family - Smoking - Use of nephrotoxic agents - Others: | | | | |
| **SUBDIMENSION 1.2: Prevention strategies for CKD in patients at risk of developing the disease.** | | | | | |
| Q2: What or what health offer (s) does the unit have for the population groups previously identified as at risk for developing Chronic Kidney Disease? | - Health education groups, name on which topics:   o Home Visits, name which professionals:  o Multiprofessional service, name which professionals:  o Drug treatment to control risk factors  o Health consultation, name which professionals:  o Other types of assistance: | | | | |
| **SUBDIMENSION 1.3: Early Diagnosis** | | | | | |
| vQ3: What or what diagnostic resource (s) do you use to identify the patient with Chronic Kidney Disease? | o Glomerular Filtration Rate (GFR) estimated by formulas based on serum creatinine o Glomerular Filtration Rate (GFR) by creatinine clearance in 24-hour urine the Urine Summary Examination (EAS) or Urine I serum creatinine o 24h Creatinine Clearance o Microalbuminuria isolated o Microalbuminuria 24h o Albuminuria Creatininuria (RAC) ratio in an isolated urine sample the US kidneys and urinary tract the urinary tract RX Tomography the other: | | | | |
| Q4: For which users are these exams requested? | | | | | - All patients - Patients considered at risk - Others |
| Q5: For at-risk individuals in whom CKD was not diagnosed in the first assessment, what is the frequency of requesting these tests in the patient's routine? | | | | | - Monthly - the Semiannual - the Annual - the Bianual - At clinical discretion - the other: |
| Q6: Do you know how long the patient takes between requesting the exam (s) and knowing your result? | | | | | - Yes, average time: - No |
| **SUBDIMENSION 1.4: Treatment** | | | | | |
| Q7: Do you attend patients with CKD in this service? | - Yes - No | | | | |
| Q8: Do you rate the stages of CKD? | - Yes - No | | | | |
| Q9: What stages do you follow exclusively in this primary care service? | - Stage 1 - Stage 2 - Stage 3A - Stage 3B - Stage 4 - non-dialysis Stage 5 (ND) - Dialysis Stage 5 (D) - o I use other criteria for monitoring, which one: | | | | |
| Q10: What or what are the objective (s) in the clinical management of patients with CKD in this PHC service? | - Control of blood glucose, - Control of arterial hypertension, - Control of dyslipidemia, - Obesity control (maintain BMI <25), - Control of cardiovascular diseases, - Smoking cessation - Adequacy of lifestyle. - Decreased sodium intake (less than 2 g / day) corresponding to 5 g of sodium chloride, in adults, unless otherwise indicated; - Encourage physical activity compatible with cardiovascular health and tolerance: 30-minute walk 5x per week - Other objectives: | | | | |
| Q11: Do you refer patients with CKD to update the vaccination calendar, according to the National Immunization Program of the Ministry of Health (PNI / MS)? | - Yes - No | | | | |
| Q12: What factors do you identify as predictors * of CKD progression?  * predictors are markers that the individual with CKD has a worse prognosis for loss of renal function throughout the clinical evolution. | - Poorly controlled blood pressure - Poorly controlled blood glucose - High cholesterol - Intensity of albuminuriaTabagismo - Use of nephrotoxic agents - More advanced stages of CKD - Others: | | | | |
| **DIMENSION 2- REGULATION OF THE PATIENT IN THE CARE NETWORK OF DRC CARRIERS.** | | | | | |
| vQ13: Do you know in your municipality if there is a protocol for referring cases that you consider necessary for the nephrologist? | | - Yes, I know and use - Yes, I know it but I don't use it - I don't know | | | |
| Q14: What cases do you refer to other points of attention? | | - Stage 1 - Stage2 - Stage 3 A - Stage 3 B - Stage 4 - non-dialysis Stage 5 (ND) - Stage 5- need to start dialysis - Other criteria for referral: | | | |
| Q15: Do you know the average time that the patient waits between referral and consultation with the nephrologist? | | - Yes, average time of:   o I don't know | | | |
| Q16: Is there integration between the PHC service and the nephrology services of the health network? | | - Yes how? check below: - The telephone - the e-mail - Electronic regulation - Regulation by reference / counter-reference on paper - Meetings between professionals from different services to discuss cases / matrix support - Other means: - No | | | |
| **DIMENSION 3- AVAILABILITY OF RESOURCES OF THE CARE LINE OF THE CARE OF CHRONIC KIDNEY DISEASE IN SUS.** | | | | | |
| Q17: Do you know the secondary and tertiary services available (specialty clinics, kidney replacement services, hospital, etc.), in your municipality, to assist patients with CKD? | | | | o Yes. Name which ones you know:       - There are no such resources in the municipality - I don't know | |
| Q18: Do you know where secondary and tertiary care resources are offered to assist patients with CKD that are not available in your municipality? | | | | - I don’t Know - Yes, in the municipality - Not applicable | |
| **DIMENSION 4: COORDINATION OF CARE FOR THE CARE OF CHRONIC KIDNEY DISEASE WITHIN THE FRAMEWORK OF PRIMARY CARE.** | | | | | |
| Q19: Does the team have access to patient information referred to other CKD monitoring services? | | | - Yes   If so, how:   - Patient report - Information System - Formal counter-reference - Other means: - No | | |
| Q20: Does the referred patient keep returning to the unit or receive interventions from team professionals? | | | - Yes. If so, in which services: - Medical consultation - Nursing consultation - Multiprofessional consultation - Health Education Groups - Pharmacy - Vaccine - Other Services: - No | | |
| Q21- Do you receive any type of professional matriculation or specialized support or consultancy from other professionals or services on the topic? | | | - o Yes - formal (by institution / municipality) - o Yes- informal through contacts with colleagues - No | | |
| Q22: Do you consider yourself fit to approach CKD in the stages attributable to PHC? | | | - Yes - No, justify: | | |
| Q23: Are you aware of the 2014 Ministry of Health Guideline on care for patients with CKD in primary care? | | | - Yes - No | | |

**QUESTION:**

Q24- Would you like to point out any difficulties in paying attention to patients with CKD in PHC?
